# Uterine inflammatory myofibroblastic tumor presented as abnormal uterine bleeding: Two cases report and literature review

**DOI:** 10.1097/MD.0000000000032141

**Published:** 2022-12-16

**Authors:** Furong Tang, Guanlin Dai, Xing Huang, Danqing Wang

**Affiliations:** a Department of Obstetrics and Gynecology, West China Second University Hospital of Sichuan University, Chengdu, Sichuan, China; b Key Laboratory of Birth Defects and Related Diseases of Women and Children (Sichuan University), Ministry of Education, Chengdu, Sichuan, China.

**Keywords:** anaplastic lymphoma kinase, diagnosis, inflammatory myofibroblastic tumor, treatment

## Abstract

Inflammatory myofibroblastic tumor (IMT) is an extremely rare soft tissue neoplasm consisting of the proliferation of fibroblastic-myofibroblastic cells with inflammatory infiltrates. It is known to occur in many parts of the body and can generally present with benign or locally recurrent behavior. Anaplastic lymphoma kinase is a specific diagnostic marker for IMT, and approximately 50% of IMT patients have anaplastic lymphoma kinase gene rearrangements. Reports of the female genital tract are rare.

**Patient concerns:**

Two patients (a 32-year-old multigravida and a 22-year-old nullipara) visited our clinic because of abnormal uterine bleeding and a uterine mass.

**Diagnoses:**

Histopathological examination, immunohistochemical markers, and fluorescence in situ hybridization confirmed the presence of a rare uterine IMT.

**Interventions:**

The masses were completely resected via hysteroscopy. The multigravida recurred rapidly in terms of symptoms and images, whereas the nullipara was complaint-free during the follow-up period. Finally, the multigravida underwent hysterectomy and bilateral salpingectomies.

**Outcomes and lessons:**

Uterine IMTs can be easily overlooked because of their extremely low incidence rate and insufficient awareness among clinicians; however, uterine IMTs need to be considered in the differential diagnosis of uterine masses. Possible differences in the biological behavior of IMT may exist in different individuals.

## 1. Introduction

Inflammatory myofibroblastic tumor (IMT) is an uncommon soft tissue neoplasm, consisting of the proliferation of fibroblastic-myofibroblastic cells with inflammatory infiltrates.^[1]^ IMT was initially described in the lung^[2]^ and previously considered as an inflammatory pseudotumor, plasma cell granuloma, or pseudosarcomatous myofibroblastic proliferation,^[3]^ but the recent identification of gene translocations involving the anaplastic lymphoma kinase (ALK) receptor tyrosine kinase at chromosome 2p23 in some IMTs proves the fact that these proliferations may be neoplasms.^[4,5]^ The etiology is unclear. IMT can appear in different body parts, but uterine localization is rare. Abnormalities of ALK occur in a significant proportion of IMT.^[6]^ In fact, morphologic features of uterine IMTs usually overlap with other mesenchymal tumors more commonly seen in the female genital tract.^[7]^ This disease may be overlooked because of insufficient awareness. Here, we report 2 cases of uterine IMT where our patients presented with abnormal uterine bleeding and uterine mass but were treated with different operation methods.

## 2. Case report

### 2.1. Case 1

One 32-year-old multigravida presented with a 1-year history of recurrent excessive menstrual bleeding. Without a history of oral contraception or a progesterone intrauterine device, the increased menstrual flow was twice as much as before. Physical and gynecological examination revealed no apparent signs. Serum tumor markers (CA-125, 17.0 U/mL [normal range, no more than 35 U/mL], carcinoembryonic antigen, 0.6 ng/mL [normal range, no more than 2.5 ng/mL], CA15-3, 11.1 U/mL [normal range, no more than 21 U/mL], CA19-9, 14.4 U/mL [normal range, no more than 34.1 U/mL]) were negative.

**Figure 1.**
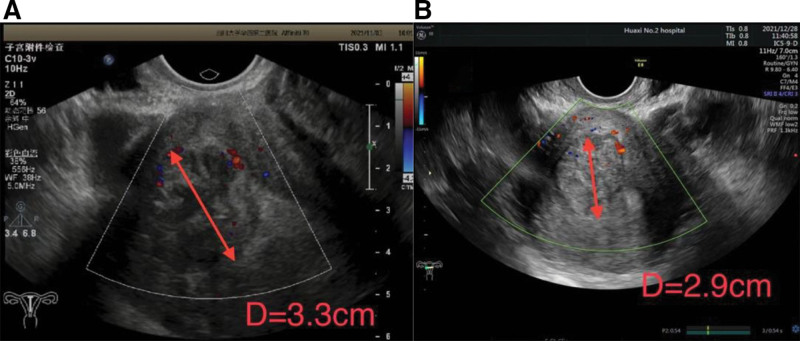
Transvaginal US image of the lesion. (A) After the first hysteroscopy surgery, a mass of heterogeneous weak echo about 3.1 × 3.3 × 2.5 cm in size with a clear border and little blood flow signal was detected in the right side of the uterine cavity. (B) After the second hysteroscopy surgery, the transvaginal US again showed a similar mass in the uterine cavity.

The past transvaginal ultrasound (US) scan had shown a submucous mass about 4cm in diameter in her uterine cavity, and she had accepted the first resection of the mass by hysteroscopy. The postoperative normal histological examination revealed leiomyoma. Menstruation recovered after the operation. However, the symptom of excessive menstrual bleeding didn’t alleviate. A transvaginal US scan for the first postoperative review after one month showed the mass still existed in her uterine cavity, with a volume of about 3.1 × 3.3 × 2.5cm (Fig. 1A).

She accepted the second hysteroscopy surgery. In the operation, a submucous myoma-like mass of about 3 cm in diameter was seen in the uterine cavity, about 1/2 of which was convex to the uterine cavity and 1/2 of which was in the muscle wall. The mass was resected almost completely in the operation. The rapid frozen pathological result in the operation was leiomyoma as well. In the postoperative histological examination, the routine immunohistochemistry test was performed which was positive for caldesmon, and FH, and especially strongly positive for ALK. As there is no ALK positivity in normal uterine tissues,^[8]^ ALK usually is seen as a specific diagnostic marker for IMTs.^[9]^ A further ALK fluorescence in situ hybridization examination has found ALK translocation in 42% of the tumor cells. All the pathologic examinations confirmed the rare uterine spindle cell neoplasm.

**Figure 2.**
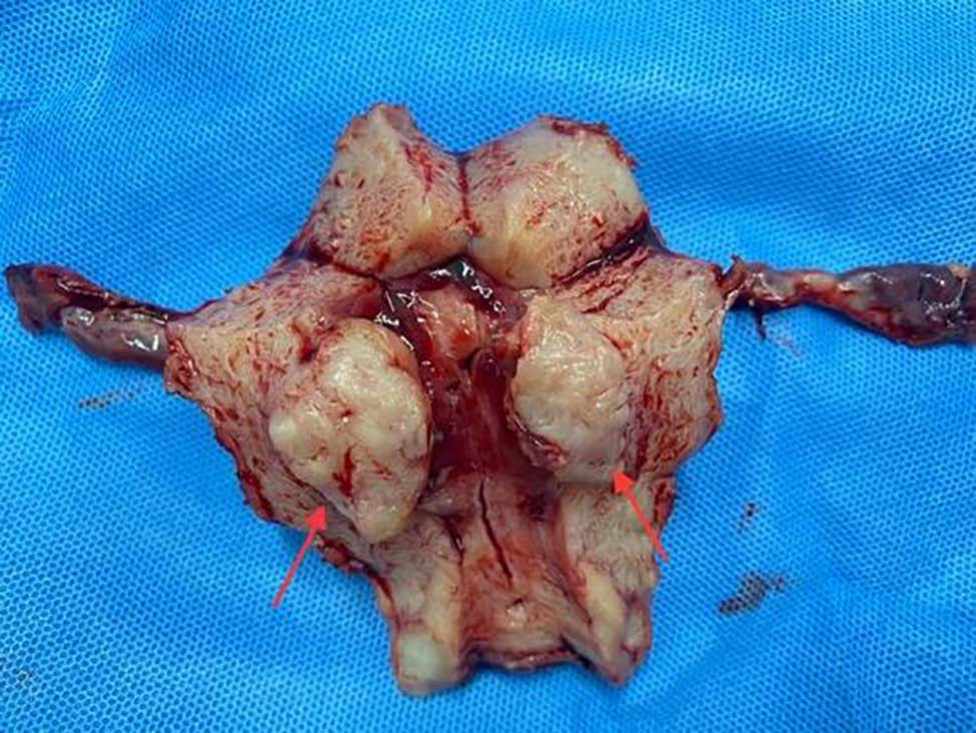
The morphologic image of the lesion. A myoma-like mass, V = 3.3cm × 2.8cm × 2.2cm, protruding toward the uterine cavity was seen in the anterior uterine myometrial wall with a seemingly clear demarcation from the surrounding area.

One month after the definite diagnosis, the symptom of excessive menstrual bleeding recurred, and the transvaginal US scan for postoperative review revealed a mass still existed in her uterine cavity (Fig.1B). Due to no significant relief of symptoms and no fertility requirements, she finally has decided to accept the hysterectomy and bilateral salpingectomy (Fig. 2).

The Immunohistochemistry of this tumor was strongly positive for ALK, which proved a local recurrence of IMT in the uterine cavity. The tumor invaded less than 1/2 whole layer of the muscle wall without lymphovascular infiltration. The tumor did not invade the cervix, surgical margins of the pelvic side walls, parametrial tissues, or fallopian tubes. In about 1 year of follow-up, she has undergone 4 times transvaginal US scans. There was no sign of local recurrence until now.

### 2.2. Case 2

**Figure 3.**
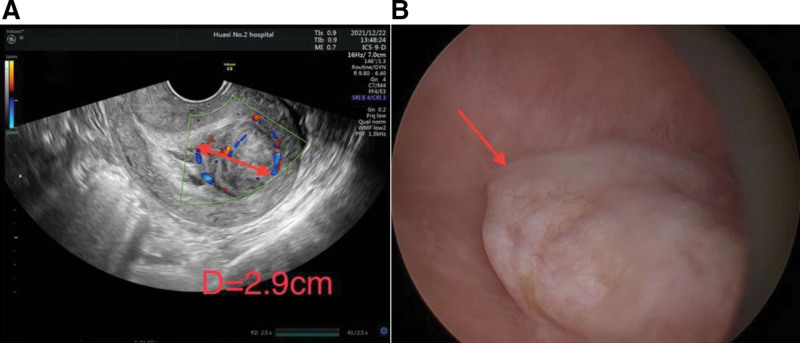
(A) Transvaginal US image of the lesion. A mass of heterogeneous weak echo with a little surrounding blood flow signal. (B) The appearance of the lesion via hysteroscopy. A myoma-like mass protruded toward the uterine cavity with few blood vessels on the surface.

One 21-year-old young woman complained of abnormal uterine bleeding with spotting in the intermenstrual period and excessive menstrual flow. The transvaginal US scan revealed a submucous myoma-like mass in her uterine cavity which gradually enlarged in size over the years (Fig. 3A).

The hysteroscopy showed a myoma-like mass about 3 cm in diameter located in the posterior wall of the uterus, with few bloodstreams on the surface and a gray section (Fig. 3B). After complete mass resection, the uterine cavity shape recovered to normal, and a normal appearance of endometrium was seen.

The histological examination combined with immunohistochemistry proved IMT, and the ALK fluorescence in situ hybridization examination found ALK translocation in 38% of the tumor cells. In the first postoperative review after one month, enhanced computerized tomography (CT) scans showed no other abnormalities such as pelvic and abdominal masses or metastasis signs. In about 1 year of the follow-up, the woman was complaint-free, and the transvaginal US scans showed no local recurrence sign.

## 3. Discussion

With uncertain etiology, IMT is a mesenchymal neoplasm with low but definite malignant potential. Its recurrence and metastasis have been recorded in about 25% and 2% of cases respectively.^[10]^ IMT involving the female genital tract is seen as a rare event, and it can affect women of all ages but predominantly reproductive-age women.

The symptoms of uterine IMT can be heterogeneous, mainly depending on the localization and size of the tumor.^[11]^ In our report, 2 patients presented with submucous myoma-like masses, resulting in abnormal uterine bleeding, while physical and gynecological examinations revealed no apparent signs due to small sizes. With rarity and nonspecific symptoms, gynecologists can hardly recognize the rare disease before performing surgery or biopsy.^[12]^

At present, the imaging methods were universally selected before surgery. Contrast-enhanced CT^[13]^ and positron emission tomography CT^[12]^ were also performed to detect activity and metastasis, while magnetic resonance imaging^[11]^ was performed for better soft tissue specificity and to avoid radiation exposure. Our chosen diagnostic imaging method was US for convenience, and inexpensiveness, and can be completed in routine medical examinations. And hysteroscopy was performed for surgery.

Because of its rarity, pathologic diagnosis is challenging as well. Pathologists need to know this rare tumor can involve various organs throughout the body.^[12]^ And when it comes to pathologic diagnosis, uterine IMT needs to differentiate from leiomyoma, leiomyosarcoma, endometrial stromal sarcoma, and fibromatosis or ligament-like fibromas^[14]^ (Table 1).

**Table 1:** The differential diagnosis of uterine IMT.^[15–19]^

	Histological features		Immunohistochemical features				Gene feature
	Major tumor tissue/cell features	Background	ALK	p16/p53	CD10	Myogenic markers	
IMT	Fibroblastic-myofibroblastic cells	plasma cells lymphocytes	+	−	+/−	desmin +/-	ALK gene fusions
						SMA +/-	
						caldesmon +/-	
LM	Smooth muscle cells	small lymphocytes	−	−	+	SMA +	NA
						desmin +	
						ER, PR+	
LMS	Tumor cells with diffuse nuclear atypia	NA	+/−	+	+/−	desmin +	PLAG1 fusions
						caldesmon+	
						SMA +/-	
LG ESS	Monotonous small blue cells	thin-walled capillaries	−	NA	+	actin +	JAZF1-SUZ12 fusions
						keratin+	
						calretinin +	
FM	Myofibroblast	collagenous matrix	−	−	NA	β-catenin +	NA

− = negative, += positive, FM = fibromatosis, IMT = inflammatory myofibroblastic tumor, LGESS = low-grade endometrial stromal sarcoma, LM = leiomyoma, LMS = leiomyosarcoma, NA = not applicable, PGR = progesterone receptor, PLAG = pleomorphic adenoma gene.

Misdiagnosis as a leiomyoma or other benign tumor is incessant, and its true morbidity may be much higher than estimated.^[20]^ The diagnosis must depend on histopathologic examination combined with immunohistochemistry. Abnormalities of ALK occur in a significant proportion of IMT,^[6]^ ALK is a specific diagnostic marker for uterine IMTs. ALK protein expression and ALK gene rearrangements were seen as powerful aids in IMT diagnoses.^[9]^

In these 2 cases, both patients were reproductive-age women without a history of oral contraception or a progesterone intrauterine device. After similar hysteroscopic resection of the lesion as far as possible, one case recurred rapidly after the hysteroscopy, while the other case had no recurrence sign with a better prognosis. ALK pathway may have played an important role in the disease.^[21]^ A phase I dose-escalation trial of the selective MET/ALK inhibitor crizotinib showed a long-term partial response in one patient with IMT who carried an ALK translocation.^[22]^ The different ALK gene rearrangement rates may contribute to the different treatment results of local mass complete resection by hysteroscope since the patient with a local recurrence has a higher ALK translocation rate. It may reveal a possible difference in the biological behaviors of IMT in different individuals.

For these 2 patients, different final therapy measures were performed individually based on age, symptom, reproductive requirement, therapy response, and local recurrence. In the following period, both patients had no sign of recurrence. However, the management of uterine IMT has never been standardized so far,^[23]^ and more cases are needed to summarize the optimal management of uterine IMT.

## Acknowledgments

This manuscript did not accept any writing or financial assistance.

## Author contributions

**Conceptualization:** Furong Tang.

**Data curation:** Furong Tang, Guanlin Dai, Xin Huang.

**Formal analysis:** Furong Tang.

**Investigation:** Guanlin Dai.

**Methodology:** Danqing Wang.

**Project administration:** Furong Tang.

**Supervision:** Danqing Wang.

**Writing – original draft:** Furong Tang.

**Writing – review & editing:** Danqing Wang.
